# Sonography versus anterior segment OCT for imaging bleb morphology after glaucoma drainage device surgery

**DOI:** 10.1007/s00417-026-07276-3

**Published:** 2026-05-20

**Authors:** Pascal Schipper, Benjamin Aretz, Leonie Bourauel, Carolin Deubel, Michael Petrak, Frank G. Holz, Raffael Liegl, Karl Mercieca, Constance Liegl

**Affiliations:** 1 Department of Ophthalmology, University of Bonn, Bonn, Germany; 2 Institute for Medical Biometry, Informatics and Epidemiology, University of Bonn, Bonn, Germany; 3 Department of Ophthalmology, Klinikum Dortmund , University Witten/Herdecke, Dortmund, Germany

**Keywords:** Glaucoma surgery, PAUL^®^ glaucoma implant, Glaucoma drainage device, Sonography, Anterior segment OCT

## Abstract

**Purpose:**

The PAUL^®^ Glaucoma Implant (PGI) is increasingly used in the surgical management of complex glaucoma, but detailed evaluation of its subconjunctival bleb morphology remains challenging due to its posterior location. While ultrasonography has long been used for bleb imaging, anterior segment optical coherence tomography (AS-OCT) offers higher-resolution imaging of anterior structures. This study aimed to directly compare ultrasonography and AS-OCT in assessing bleb morphology following PGI implantation.

**Methods:**

In this prospective observational study, 20 patients (21 eyes) with PGI underwent postoperative assessment with both AS-OCT and B-scan ultrasonography. Bleb configuration (single- vs. double-layered), bleb height, and spatial extension were evaluated and compared between modalities.

**Results:**

Mean age was 64.0 years (SD = 16.6); 80% were male. Sonography identified double-layered blebs in 95.2% of eyes, compared to 47.7% with AS-OCT, which failed to fully visualize bleb layers in 38.1% of eyes. Sonography consistently measured greater bleb heights and lengths than AS-OCT (total bleb height 3.76 ± 1.39 mm vs. 1.77 ± 0.71 mm, *p* < 0.001; maximum bleb length 17.24 ± 3.17 mm vs. 2.85 ± 1.03 mm, *p* < 0.001).

**Conclusions:**

Sonography provided more comprehensive visualization of PGI blebs, particularly in detecting double-layered configurations, total height and measuring full posterior extension, whereas AS-OCT offered detailed anterior segment assessment including the tube entry site and patch graft. Both modalities yield complementary information, and their combined use may enhance postoperative monitoring and early detection of bleb dysfunction.

## Key messages

**What is known:**

Imaging of glaucoma drainage device (GDD) blebs is challenging due to their posterior location.

Ultrasonography and anterior segment OCT (AS-OCT) are both used in postoperative GDD assessment, but their relative performance for PAUL® Glaucoma Implant (PGI) bleb visualization has not been directly compared.

**What is new:**

Sonography provides more comprehensive visualization of PGI blebs, including posterior extension and detection of double-layered bleb configurations.

AS-OCT reliably visualizes anterior structures such as the tube entry site and patch graft but consistently underestimates total bleb dimensions compared with sonography.

The two modalities offer complementary information; combining sonography and AS-OCT may enhance postoperative monitoring and early detection of bleb dysfunction.

## Introduction

**Fig. 1:**
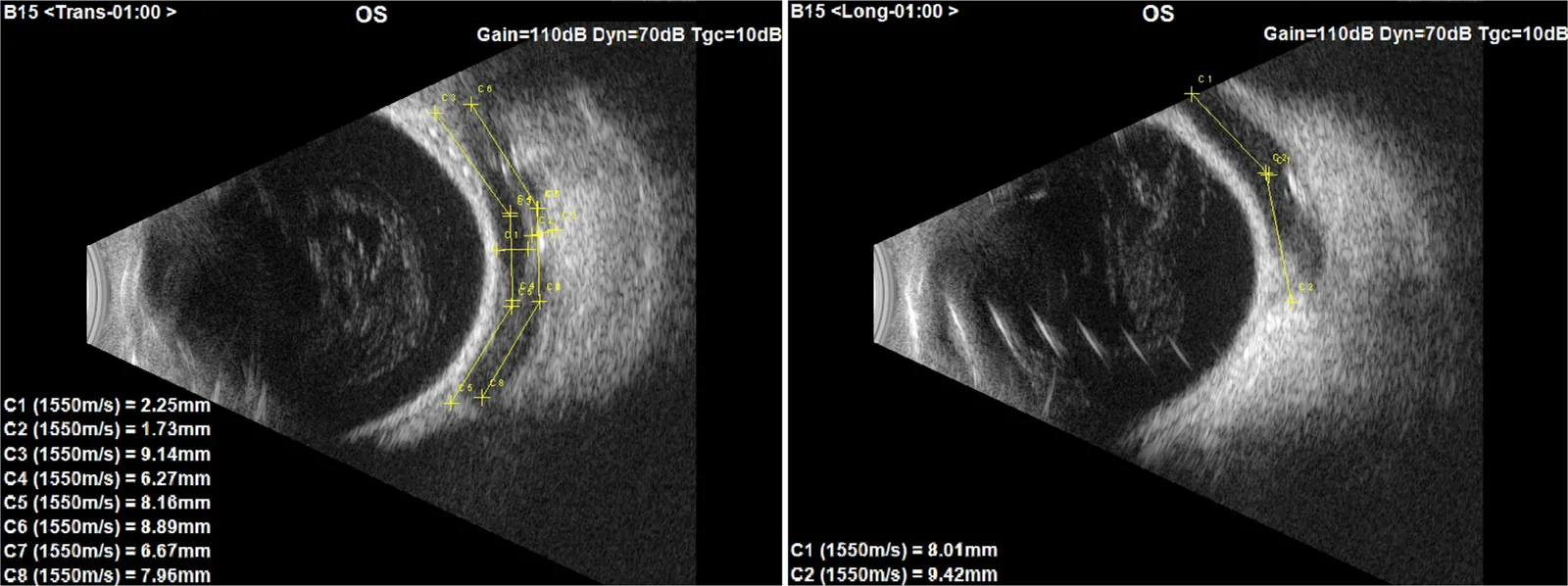
Bleb measurement in both transverse (T) and longitudinal (L) sections and configuration of bleb morphology (one or two compartments), in this case with double-layer

The surgical management of glaucoma has advanced considerably in recent years, with glaucoma drainage devices (GDD) becoming a cornerstone in the treatment of complex or refractory cases [1–3]. Among the available implants, the PAUL^®^ Glaucoma Implant (PGI) represents the latest addition to the armamentarium, combining a large plate surface area with a smaller tube diameter, aiming to achieve effective intraocular pressure (IOP) control while minimizing the risk of hypotony and other complications [4, 5]. Long-term success of GDD surgery depends largely on the function of the subconjunctival filtering bleb that forms around the implant plate. However, due to its posterior and deep location, traditional slit-lamp biomicroscopy provides limited insight into bleb morphology. Although magnetic resonance imaging has been used to characterize GDD blebs and has demonstrated associations between bleb configuration and IOP outcomes [6–10], its high cost, limited accessibility, and lack of routine clinical feasibility limit its use. Ultrasonography, in contrast, is more practical and widely available, and a few studies have described its utility in visualizing GDD blebs [11–13]. Anterior segment optical coherence tomography (AS-OCT) offers high-resolution imaging of anterior ocular structures and has recently emerged as a promising tool in postoperative GDD assessment. It has already proven effective in evaluating patch graft integrity and detecting early signs of tube exposure risk [7–9]. Despite this potential, no studies to date have systematically compared AS-OCT with ultrasonography for the visualization and characterization of GDD blebs (Fig. 1).

**Fig. 2:**
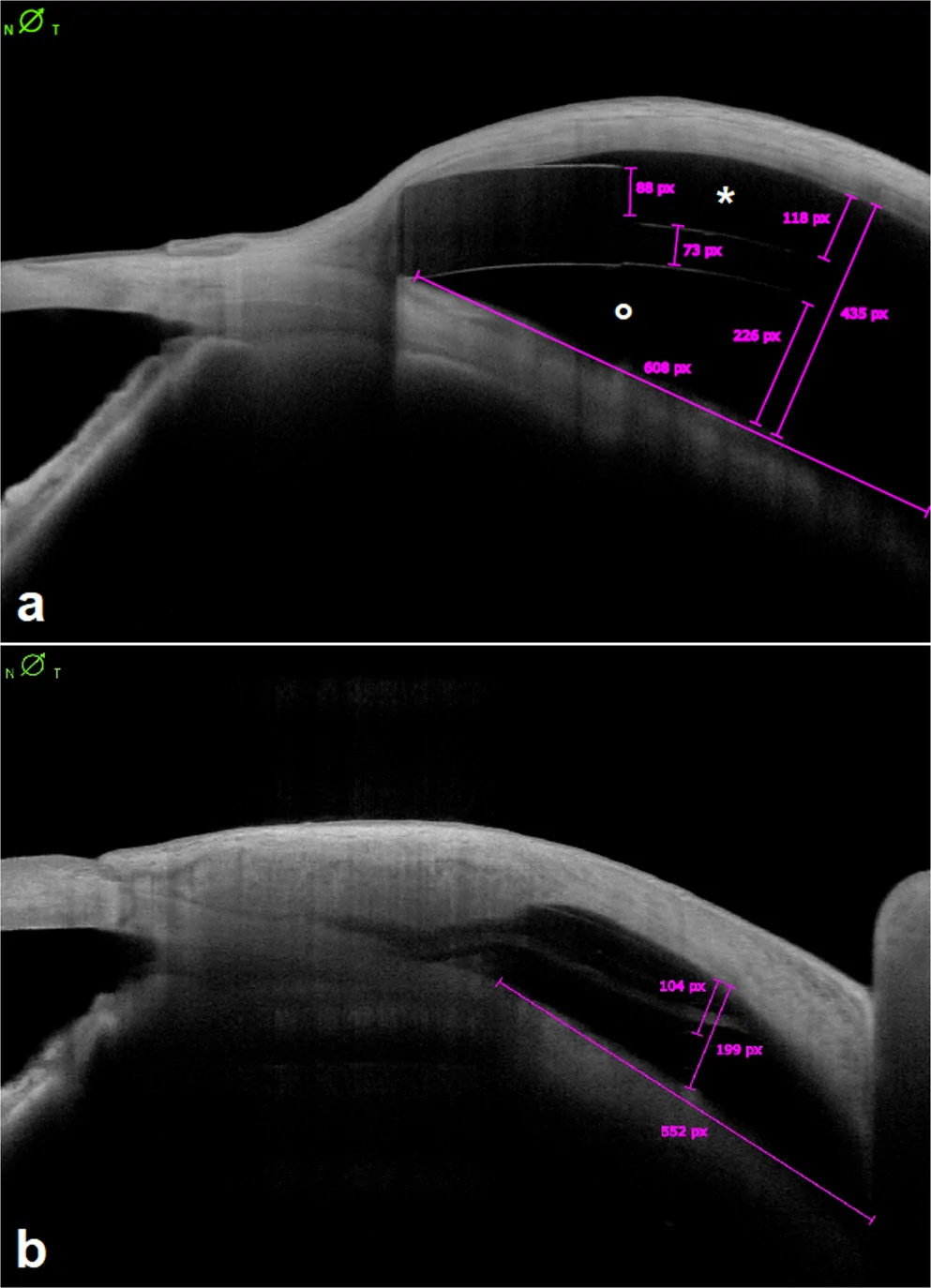
Example measurement of bleb parameters using high-resolution AS-OCT. Part a shows a double-layer bleb. * indicates the superior compartment (with a width of 118 px) and ° indicates the inferior compartment (with a width of 226 px). Part b shows a single-layer bleb in which only a small superior compartment with a width of 104 px is visible. The intraluminal Prolene thread is also visible here. The values in pixel (px) were converted in µm and mm for the statistical analysis

This study aims to address this gap by directly comparing AS-OCT and ultrasonography in assessing filtering blebs following PGI implantation. By evaluating imaging quality and bleb morphology, we aim to determine the relative strengths and limitations of each modality and explore their potential roles in postoperative management of GDD (Fig. 2).

## Materials and methods

### Study design and participants

This prospective observational study included patients who presented for postoperative follow-up after PAUL^®^ Glaucoma Implant (PGI) surgery at the glaucoma service of the University Eye Hospital Bonn, Germany, between January and May 2024. All participants underwent both high-resolution anterior segment optical coherence tomography (AS-OCT; ANTERION^®^, Heidelberg Engineering, Germany) and standardized B-scan ultrasonography using a 15 MHz probe (Compact Touch, Quantel Medical, France) at the respective follow-up time point. Both imaging modalities were used to assess bleb morphology. Specifically, measurements of bleb height and extension were obtained, and the presence of a single- versus double-compartment bleb configuration was documented. A full ophthalmological examination was conducted during each visit, including best-corrected visual acuity (BCVA) using Snellen charts (converted to logMAR), intraocular pressure (IOP) via Goldmann applanation tonometry, slit-lamp biomicroscopy, and fundus examination.

Patient medical records were reviewed to obtain preoperative demographics and clinical characteristics such as age, gender, glaucoma subtype, BCVA, IOP, and anterior segment findings. Postoperative data included longitudinal records of BCVA, IOP, number of topical IOP-lowering agents, any complications, and changes in therapy.

The primary objective of the study was to compare AS-OCT and sonography in terms of bleb visualization—specifically evaluating bleb height, extension, and compartmentalization. Secondary objectives included analysis of IOP, BCVA, and number of medications. Ethical approval was obtained from the institutional review board of the University Hospital Bonn (approval number:2024-17-BO & 091/22), and the study complied with the principles of the 2024 Declaration of Helsinki.

## Image acquisition and processing

AS-OCT imaging was performed by trained ophthalmic technicians following a standardized scanning protocol. Each scan series consisted of 25 radial sections, captured at an angle of 45° or 135°, depending on implant location. Images spanned 16.0 mm in length and 7.5 mm in height. All scans were conducted on the same AS-OCT system (Heidelberg ANTERION^®^ Swept-Source OCT, Heidelberg Engineering GmbH, Germany). The section with the greatest expansion of the bleb was selected and quantitative analysis was performed using Heidelberg Eye Explorer software (HEYEX2), with manual calliper tools used to obtain measurements.

To ensure consistent measurement of patch thickness and bleb morphology, the reference point for the measurements was defined as the highest visible bleb height in the respective scan. A pixel-to-micrometre conversion factor of 6.82 was applied, based on prior internal calibration using Pentacam^®^ (OCULUS Optikgeräte GmbH, Germany) data and validated AS-OCT reference measurements.

To assess the filtering bleb, both longitudinal and transverse sonographic scans were performed. However, only transverse images were used for direct comparison with AS-OCT, as they provided a spatial orientation consistent with the OCT scan planes.

The imaging protocol and measurements used in this study were internally validated for reproducibility and consistency. Prior work by the research group has demonstrated high inter- and intra-observer agreement using this protocol [14].

### Statistical analysis

All analyses were performed using SPSS version 27.0 (IBM Corp., Armonk, USA). Descriptive statistics were used to summarize the cohort: continuous variables were expressed as mean, standard deviation, and range; categorical variables as absolute counts and percentages. Continuous paired measurements of bleb dimensions obtained by sonography and AS-OCT in the same eyes were compared using a paired-samples t-test when the distribution of differences was approximately normal (assessed with the Shapiro–Wilk test) or with the Wilcoxon signed-rank test when normality was not met. Results are reported as mean ± standard deviation, and two-sided p-values < 0.05 were considered statistically significant.

For paired categorical outcomes—specifically, the presence of single-, double-layered, or unidentifiable blebs—differences between the two imaging modalities were evaluated using the chi-square test.

All statistical tests were exploratory in nature, two-sided, and a p-value < 0.05 was considered statistically significant.

## Results

Twenty patients (21 eyes) who underwent PGI surgery were included in the study. Sixteen patients were male and four were female. The mean age was 64.0 years (SD = 16.6). The mean follow-up time at the time of the respective investigation was 19.6 months (SD = 8.5). Diagnoses included primary open-angle glaucoma (POAG) in 9 eyes, pseudoexfoliation glaucoma in 3 eyes, and secondary glaucoma in 9 eyes. Seven patients had undergone previous pars plana vitrectomy, and 15 eyes had at least one prior glaucoma surgery. Seventeen eyes were pseudophakic, two were phakic, and two aphakic. Mean pre-operative IOP was 24.0 mmHg (SD = 7.7) and decreased to 12.2 mmHg (SD = 3.7) at the respective postoperative follow-up time point. The mean number of pre-operative glaucoma medications was 3.4 (SD = 1.1), which was reduced to 0.7 (SD = 0.9) post-operatively. While 95.2% of eyes required glaucoma drops pre-operatively, only 42.6% did so following PGI surgery. The mean pre-operative BCVA was 0.5 logMAR (SD = 0.6), which decreased to 0.7 logMAR (SD = 0.7) post-operatively. Further demographic and clinical characteristics are shown in Table 1.

**Table 1:** Demographics and clinical characteristics of patients undergoing PGI surgery

Gender	*n* = 21 eyes, 20 patients
Male/female (%)	16 (80)/4 (20)
Ethnicity	
Caucasian (%)	19 (95)
Other (%)	1 (5)
Age	
Mean (SD)	64.0 (16.6)
Glaucoma type	
Primary open-angle glaucoma (%)	9 (42.9)
Pseudoexfoliation glaucoma (%)	3 (14.3)
Narrow-angle glaucoma (%)	0 (0)
Secondary glaucoma (%)	9 (42.9)
Previous vitrectomy	
Yes (%)	7 (33.3)
Previous glaucoma surgery	
Yes (%)	15 (71.4)
Which surgery	
Trabeculectomy/revision (%)	7 (33.3)/2 (9.5)
Preserflo (%)	1 (4.8)
Trabectome/Kahook (%)	2 (9.5)/1 (4.8)
CPC (%)	4 (19.0)
Deep sclerectomy (%)	1 (4.8)
XEN (%)	2 (9.5)
Number (#) of glaucoma drops pre-operative	
Mean (SD)	3.4 (1.1)
Number (#) of glaucoma drops post-operative	
Mean (SD)	0.7 (0.9)
Glaucoma drops pre-operative	
Yes (%)	20 (95.2)
Glaucoma drops post-operative	
Yes (%)	9 (42.6)
Lens status	
Phakic/pseudophakic/aphakic (%)	2 (9.5)/17 (81.0)/2 (9.5)
BCVA pre-operative (logMAR)	
Mean (SD)	0.5 (0.6)
BCVA post-operative (logMAR)	
Mean (SD)	0.7 (0.7)
IOP pre-operative (mmHg)	
Mean (SD)	24.0 (7.7)
IOP post-operative (mmHg)	
Mean (SD)	12.2 (3.7)

As shown in Table 2, postoperative bleb morphology differed significantly between ultrasound and AS-OCT examinations. Sonography identified single-layered blebs in 1 eye (4.8%) and double-layered blebs in 20 eyes (95.2%). In contrast, AS-OCT identified single-layered blebs in 3 eyes (14.3%), double-layered blebs in 10 eyes (47.7%), and non-identifiable bleb layers in 8 eyes (38.1%). Quantitative measurements were consistently larger with sonography than with AS-OCT: Superior bleb height was greater when measured by sonography (mean = 1.65 mm, SD = 0.43, *n* = 21) compared to AS-OCT (mean = 0.39 mm, SD = 0.24, *n* = 13). Inferior bleb height also differed, with mean values of 2.21 mm (SD = 1.07, *n* = 20) on sonography versus 0.75 mm (SD = 0.46, *n* = 10) on AS-OCT. The total bleb height was significantly higher on sonography (mean = 3.76 mm, SD = 1.39, *n* = 21) than on AS-OCT (mean = 1.77 mm, SD = 0.71, *n* = 21). The maximum visible bleb length averaged 17.24 mm (SD = 3.17) on sonography compared to 2.85 mm (SD = 1.03) on AS-OCT.

**Table 2:** Comparison of post-operative bleb measurements between sonography and AS-OCT

	Sonography	AS-OCT	*P*-value
Single-layer bleb (%)	1 (4.8)	3 (14.3)	0.317^1^
Double-layered bleb (%)	20 (95.2)	10 (47.7)	
Bleb layers not fully identifiable (%)	0 (0)	8 (38.1)	
Superior bleb height in mm (SD; n)	1.65 (0.43; 21)	0.39 (0.24; 13)	< 0.001^2^
Inferior bleb height in mm (SD; n)	2.21 (1.07; 20)	0.75 (0.46; 10)	< 0.001^2^
Total height in mm (SD; n)	3.76 (1.39; 21)	1.77 (0.71; 21)	< 0.001^2^
Maximum visible bleb length in mm (SD; n)	17.24 (3.17; 21)	2.85 (1.03; 21)	< 0.001^2^

^1^ Chi-square test; ^2^ Wilcoxon signed-rank test

When comparing postoperative bleb characteristics between modalities, sonography consistently measured significantly greater superior, inferior, and total bleb heights as well as maximum visible bleb length (all *p* < 0.001). The proportion of identifiable bleb layers differed between techniques (*p* = 0.002), with sonography showing a higher detection rate of double-layered blebs, whereas AS-OCT more frequently failed to fully identify bleb layers.

## Discussion

This prospective study compared sonography and AS-OCT for imaging bleb morphology following PGI implantation. Both imaging modalities yielded clinically meaningful information about bleb structure and function, though with distinct differences in sensitivity and anatomical reach.

Based on our results, sonography proved particularly effective in detecting bleb configuration [11, 13], detecting double-layered blebs in 95% of eyes compared with only 48% by AS-OCT. Double-layered morphology was clinically meaningful, as it correlated with a 4.9 mmHg lower postoperative IOP after adjustment for preoperative pressure (*p* = 0.001). Sonography also provided markedly larger measurements of bleb dimensions—mean total height 3.76 mm and maximal length 17.2 mm versus 1.77 mm and 2.85 mm on AS-OCT (all *p* < 0.001)—reflecting its ability to insonate the entire posterior reservoir and identify the point of maximum height. These capabilities make sonography particularly suited for longitudinal follow-up, assessment before intraluminal stent removal, and detection of early encapsulation.

AS-OCT, while limited in posterior imaging depth, still identified bleb compartmentalization in many cases. This is noteworthy, as until now, layered bleb configurations have primarily been described in studies using sonography or MRI [7, 8]. MRI has revealed key morphological features such as double-layered blebs that may be associated with better bleb function and lower IOP values. Other MRI studies have also shown that larger filtering blebs are associated with lower IOP values. However, MRI is expensive, time-consuming, and impractical for routine clinical use. Our findings indicate that, with careful technique, AS-OCT can detect structural features relevant to bleb function, even though it consistently underestimated bleb height compared with sonography (mean difference ≈ 2 mm), likely because it captures a more anterior segment of the reservoir. Even with downward gaze during imaging, AS-OCT cannot reliably depict the most posterior portion of the bleb, which after PGI surgery frequently extends beyond the equator.

These limitations are consistent with the anatomical drainage characteristics of GDDs, which are designed to channel aqueous toward the equatorial or post-equatorial subconjunctival space. While AS-OCT has proven effective in evaluating bleb morphology after trabeculectomy—where the bleb is more anterior and readily accessible [15, 16]—it is less well suited for visualizing the full extent of posteriorly located blebs in GDD procedures.

While MRI and sonography have provided most of the current knowledge on bleb configuration and volume, our findings suggest that AS-OCT may also play a role in longitudinal bleb monitoring. We did not assess serial AS-OCT measurements in this study, yet it is reasonable to assume that—despite consistently lower absolute height values caused by its restriction to the anterior portion of the bleb—AS-OCT could still track relative changes over time in a manner analogous to sonography. Nevertheless, sonography remains the more comprehensive modality for true longitudinal assessment, as it allows full thickness insonation of the bleb, identification of the point of maximal height, and repeated measurement of this maximum in follow-up examinations. An advantage of AS-OCT on the other hand its higher axial resolution and ability to visualize tissue microstructure offering the potential to evaluate changes in bleb wall reflectivity. Investigating whether reflectivity patterns correlate with IOP trends or bleb function over time could provide a valuable non-invasive biomarker for early bleb failure. In the present study we did not analyse bleb reflectivity, but future work could investigate whether reflectivity patterns correlate with IOP trends or bleb function over time to provide a valuable non-invasive biomarker for early bleb failure. A study of 52 eyes after Ahmed glaucoma valve (AGV) implantation demonstrated that AS-OCT can track bleb-wall thickness and reflectivity over time, showing a U-shaped thickness curve and an inverted U-shaped reflectivity pattern peaking at one month. Reflectivity was significantly higher in eyes with a hypertensive phase (IOP > 21 mmHg within three months), suggesting a link to fibrotic remodelling [17]. In another cross-sectional series of 76 AGV eyes, both maximum and minimum bleb-wall thickness were lower in successful surgeries and correlated with IOP and medication use, while maximum thickness remained an independent predictor of surgical success. Moreover, AS-OCT offers unique advantages in anterior segment imaging. It allows for detailed visualization of the tube entry site, tube-cornea distance, and overlying patch graft, which are critical for assessing risk of complications such as corneal touch or tube erosion, and for monitoring conjunctival integrity postoperatively. The results underscore that AS-OCT-derived structural parameters can serve as predictors of bleb function and outcomes and support its broader use in postoperative monitoring of GDD [18]. In practices without access to sonography, AS-OCT can still serve as a valuable imaging tool for partial bleb assessment, particularly when combined with clinical data and IOP monitoring.

To our knowledge, this is also the first study to directly compare its findings to those obtained by sonography. While AS-OCT does not reliably visualize the entire posterior bleb extent, it is still capable of detecting relative changes in bleb height over time. A progressive decrease in height, either on AS-OCT or sonography, may serve as an early indicator of fibrosis or encapsulation, prompting timely intervention.

Considering these findings, we propose that sonography and AS-OCT serve complementary roles in the postoperative evaluation of GDD blebs. Sonography offers a more complete and sensitive assessment of bleb configuration and extension, particularly in the early phases when bleb morphology is still evolving. Further, it can be used to assess bleb function before removing an intraluminal prolene stent to reduce the risk of associated hypotony. AS-OCT enhances anterior segment surveillance and may be especially useful for monitoring changes as patch material thinning or tube positioning. A multimodal imaging approach using both techniques may provide the most comprehensive and individualized patient care.

This study has several limitations that should be considered when interpreting the findings. First, the number of eyes included in our analysis was relatively small, which may limit the generalizability of our conclusions and reduce statistical power. Second, our cohort consisted exclusively of eyes implanted with the PGI. While this ensured a uniform surgical approach and minimized variability due to implant type, it also restricts the applicability of our findings to other glaucoma drainage devices, such as the Ahmed^®^ or Baerveldt^®^ implants, which differ in design, plate material, and flow dynamics. Additionally, our study was cross-sectional in design, capturing bleb morphology at a single postoperative time point. This limits our ability to assess temporal changes in bleb configuration, height, or function, and to determine whether certain imaging characteristics might predict long-term outcomes or early failure. Although we identified imaging features associated with IOP at the time of examination—such as double-layered bleb configuration and greater bleb height—these associations would need to be confirmed in prospective studies with longitudinal follow-up. A time-series analysis would be particularly valuable in evaluating how blebs evolve after surgery, how imaging parameters like height and reflectivity change over time, and whether early morphological features correlate with later surgical success or failure. Moreover, we included the mean follow-up time at the respective time of investigation, however, due to the heterogeneity in the time intervals between surgery and the measurements, we did not include exact postoperative time points for each eye and no further stratification into specific postoperative intervals was performed, as this would have resulted in small subgroup sizes with insufficient statistical power. Importantly, our analysis focuses on the comparison between two measurement methods obtained at the same visit for each eye. Therefore, the variability in postoperative timing does not materially affect the primary objective of the study, which is the agreement/difference between these methods at a given time point. Future studies with larger cohorts and longitudinal follow-up at predefined intervals are planned to further evaluate temporal changes.

Future studies should include larger and more diverse patient populations, different types of GDDs, and serial imaging at standardized time points to clarify how postoperative events such as stent removal, needling, or wound modulation influence bleb morphology and function. Combining clinical outcomes with longitudinal sonographic and AS-OCT data may enable predictive models for bleb performance and early detection of dysfunction. In addition, optimizing AS-OCT for deeper posterior imaging and developing automated tools for detecting layered structures could strengthen its role as a practical follow-up modality, especially in settings without access to ultrasound.

## Conclusion

In summary, this study demonstrates that ultrasonography and AS-OCT provide complementary insights into bleb morphology following PGI implantation. Sonography offers superior visualization of the full bleb configuration, particularly in detecting layered structures and assessing posterior extension, while AS-OCT excels in high-resolution anterior segment imaging and evaluation of tube-related complications. A multimodal approach integrating both techniques may therefore enhance postoperative monitoring, guide timely interventions, and support individualized patient care.

## Data Availability

All datasets generated during and/or analysed during the current study are available from the corresponding author on reasonable request.

## References

[1] Aref AA, Gedde SJ, Budenz DL (2017) Glaucoma Drain Implant Surg Dev Ophthalmol 59:43–52 10.1159/000458485 28442686

[2] Pereira ICF et al (2021) Conventional glaucoma implants and the new MIGS devices: a comprehensive review of current options and future directions. Eye (Lond) 35(12):3202–3221 10.1038/s41433-021-01595-x PMC8602385 34127842

[3] Lee RMH et al (2020) Translating Minimally Invasive Glaucoma Surgery Devices. Clin Transl Sci 13(1):14–25 10.1111/cts.12660 PMC6951459 31568666

[4] Tan MCJ et al (2024) Three-Year Outcomes of the Paul Glaucoma Implant for Treatment of Glaucoma. J Glaucoma 33(7):478–485 10.1097/IJG.0000000000002369 PMC11210944 38506749

[5] Weber C et al (2024) Two-year clinical outcomes of the PAUL® glaucoma implant in White patients with refractory glaucoma. J Glaucoma. 33(10):808–814. 10.1097/ijg.0000000000002457 38940658

[6] Vallabh NA et al (2021) Surgical technique, perioperative management and early outcome data of the PAUL^®^ glaucoma drainage device. Eye. 36:1905–1910. 10.1038/s41433-021-01737-1 PMC8450714 34545206

[7] Correia Barão R et al (2023) Bleb morphology of glaucoma drainage devices on magnetic resonance imaging. Acta Ophthalmol 101(7):789–796 10.1111/aos.15668 37066864

[8] Iwasaki K et al (2017) Evaluation of Bleb Fluid After Baerveldt Glaucoma Implantation Using Magnetic Resonance Imaging. Sci Rep 7(1):11345 10.1038/s41598-017-11054-x PMC5595874 28900148

[9] Sano I et al (2015) Assessment of Filtration Bleb and Endplate Positioning Using Magnetic Resonance Imaging in Eyes Implanted with Long-Tube Glaucoma Drainage Devices. PLoS ONE 10(12):e0144595 10.1371/journal.pone.0144595 PMC4671622 26641251

[10] Anderson DM et al (2017) Magnetic resonance imaging characteristics of a Baerveldt glaucoma implant. J Glaucoma 26(6):534–540 10.1097/IJG.0000000000000658 28333893

[11] Lloyd MA et al (1993) Echographic evaluation of glaucoma shunts. Ophthalmology 100(6):919–927 10.1016/s0161-6420(93)31553-8 8510907

[12] Duan X, Jiang Y, Liu X (1997) [Ocular echographic evaluation of human aqueous drainage implant]. Zhonghua Yan Ke Za Zhi 33(6):421–424 10680542

[13] Weber C et al (2024) Sonographic bleb visualisation after PAUL glaucoma implant surgery. Br J Ophthalmol. 10.1136/bjo-2024-326168 39689979

[14] Schipper P et al (2025) Anterior segment OCT for imaging PAUL^®^ glaucoma implant patch grafts: a useful method for follow-up and risk management. Graefes Arch Clin Exp Ophthalmol 263(4):1071–1079 10.1007/s00417-024-06708-2 PMC12095395 39681685

[15] Singh M et al (2007) Imaging of trabeculectomy blebs using anterior segment optical coherence tomography. Ophthalmology 114(1):47–53 10.1016/j.ophtha.2006.05.078 17070581

[16] Singh M et al (2007) Anterior segment optical coherence tomography imaging of trabeculectomy blebs before and after laser suture lysis. Am J Ophthalmol 143(5):873–875 10.1016/j.ajo.2006.12.001 17452175

[17] Jung KI et al (2015) Serial changes in the bleb wall after glaucoma drainage implant surgery: characteristics during the hypertensive phase. Acta Ophthalmol 93(4):e248–e253 10.1111/aos.12571 25363490

[18] Jung KI et al (2013) Visualization of blebs using anterior-segment optical coherence tomography after glaucoma drainage implant surgery. Ophthalmology 120(5):978–983 10.1016/j.ophtha.2012.10.015 23347982

